# The invisible backpack: how push-pull factors drive healthcare students’ cross-cultural learning attitudes and career identity

**DOI:** 10.3389/fpsyg.2026.1687038

**Published:** 2026-02-25

**Authors:** Lin Jiayin, Naif Mohammed Al-Hada

**Affiliations:** 1 Development Planning Center, Baise University, Baise, China; 2 School of Materials Science and Engineering, Baise University, Baise, China

**Keywords:** attitude formation, healthcare education, international students, push-pull factors, student mobility

## Abstract

Studying abroad has become an increasingly significant pathway for students seeking academic, professional, and personal growth in a globalized world. This review paper explores the dynamic relationship between push-pull factors and international students’ attitudes toward studying abroad, with a particular focus on the motivations and barriers that shape these perceptions. By synthesizing findings from existing literature and enrollment trends, the study identifies key factors influencing students’ decisions to pursue education abroad. Push factors, such as limited educational opportunities and socio-economic challenges in students’ home countries, are shown to significantly drive the decision to study abroad, while pull factors such as the prestige of foreign institutions and enhanced career prospects positively influence attitudes. Special attention is given to the context of healthcare education, where international training can address workforce shortages and promote cultural competency among practitioners. The findings offer valuable insights for educational policymakers and institutions, emphasizing the importance of understanding these motivational dynamics to attract and retain international students in healthcare disciplines. The paper concludes by proposing strategies that could facilitate global mobility and collaboration in healthcare education, ultimately contributing to better healthcare outcomes worldwide.

## 1 Introduction

The phenomenon of studying abroad has become a focal point of interest for educators, policymakers, and researchers, particularly as globalization continues to transform the landscape of higher education (Roy et al., 2019). International student mobility (ISM) has emerged as a crucial aspect of this transformation, driven by an intricate web of factors that both encourage students to seek educational opportunities abroad and compel them to leave their home countries (Cankaya, 2024). These factors can broadly be categorized into “push” and “pull” forces. Push factors are often related to economic, social, and political challenges in the home country, such as limited academic options, unstable governance, or lack of job prospects (Khanam and Joshi, 2025a). In contrast, pull factors are linked to the perceived advantages of studying in foreign institutions, such as better educational quality, improved career opportunities, and enhanced cultural exposure (Luo et al., 2023). This duality is commonly explored through the push-pull framework, which offers a widely used theoretical lens for understanding the motivations behind international student mobility (Mazzarol and Soutar, 2002; Cankaya, 2024).

Despite the considerable attention given to the push-pull factors in the literature, many international students report experiencing a complex mix of motivations and reservations about their decision to study abroad. These conflicting sentiments, ranging from excitement to anxiety, suggest that the decision to study overseas is not always straightforward, and the interplay between push and pull factors is far more nuanced than previously understood. This gap in the literature presents an important opportunity for further investigation. As international student mobility continues to increase globally, understanding the underlying dynamics that shape students’ attitudes and behaviors is crucial for developing policies and strategies that support their success in the host country and beyond (Ke et al., 2022; Liu et al., 2018; Wang and Crawford, 2021; Zhao et al., 2025). This review paper explores the complex push-pull factors influencing international students’ attitudes toward studying abroad. By synthesizing both qualitative and quantitative research from existing published research, it offers a comprehensive analysis of how these factors shape students’ decision-making processes. Understanding these motivations is crucial for universities aiming to attract and retain diverse international student populations, ensuring they remain competitive in the global educational landscape (Luo et al., 2023). The paper highlights the importance of context when examining motivations for studying abroad. For example, in healthcare education, international training opportunities help address workforce shortages and enhance cultural competency among practitioners. The implications of push-pull factors are particularly significant in fields like healthcare, where cross-border education can have far-reaching effects on global healthcare systems and care quality (Raju et al., 2023). These insights can guide universities and policymakers in developing strategies that attract international students while ensuring their successful integration and retention. This research is particularly timely as global demand for talent and skills shifts. As countries work to meet economic and social goals, understanding international students’ decision- making processes is essential for fostering successful educational exchanges. Educational institutions must adapt to these demands by offering environments that are welcoming, supportive, and conducive to academic and personal success (Gupta and Fernandez-Crehuet, 2021). Policymakers must consider factors such as financial assistance, visa regulations, and cultural integration when designing programs that facilitate international student mobility. Additionally, the motivations behind studying abroad vary across student demographics, influenced by socio-economic status, academic discipline, cultural background, and geographic location. This paper delves into these variations, offering a more nuanced understanding of how push-pull dynamics shape different groups’ readiness and willingness to study abroad (Rosli et al., 2024; Rosli, 2024). By examining the diverse experiences of international students, the review sheds light on the broader socio-cultural, economic, and educational factors that guide their decisions. The findings aim to inform the development of strategies that enhance student recruitment, retention, and satisfaction, fostering a more inclusive higher education system. Understanding the push-pull dynamics that influence international students’ attitudes will help institutions design programs that align with student needs, improving academic outcomes and engagement (Cai et al., 2023). This review emphasizes the importance of adapting to the diverse needs of student populations to promote a more equitable and globalized academic environment. The implications extend beyond individual decisions, suggesting that universities and policymakers should collaborate to create frameworks that attract international students while supporting their academic and personal growth throughout their educational journey. Further exploration of these dynamics will contribute to efforts aimed at enhancing global educational exchanges, fostering international collaboration, and improving the overall quality of higher education worldwide (Nikou and Luukkonen, 2023). As educational paradigms continue to evolve, understanding the push-pull factors that influence international students will be crucial for shaping the future of higher education (see Table 1).

**Table 1:** Influencing factors for international students’ decision to study abroad (Mazzarol and Soutar, 2002; Nikou et al., 2023).

Factor	Taiwan (China)	India	China	Indonesia
Overseas course better than local	92%	93%	62%	92%
Difficult to gain entry at home	59%	47%	39%	49%
Course not available at home	51%	47%	33%	51%
Better understanding of West	91%	47%	91%	80%
Intention to migrate	43%	59%	38%	40%

## 2 Literature review

In an era of accelerating globalization, student mobility across borders has become a defining feature of modern higher education. As students increasingly pursue educational opportunities abroad, their motivations are shaped by a complex web of push-pull factors. These factors, both external and internal, drive students to explore international education options, creating a nuanced decision-making process. Push factors arising from challenges in students’ home countries, such as economic instability, political unrest, and limited academic opportunities compel students to seek better prospects abroad. In contrast, pull factors such as superior academic programs, high-quality living conditions, and promising post-graduation opportunities draw students to specific host countries (Khanam and Joshi, 2025a; Zhao et al., 2025). Understanding these dynamics is crucial not only for enhancing institutional policies but also for fostering intercultural exchanges that have profound implications on global educational landscapes (Luo et al., 2023).

The significance of international education extends far beyond individual career prospects. It influences the socio-economic development of both home and host countries (Raju et al., 2023). As universities strive to attract international talent, it becomes vital to explore the diverse motivations behind students’ decisions to study abroad. The existing literature highlights a variety of personal and contextual motivations, from aspirations for personal growth to external pressures such as political and economic uncertainties in students’ home countries (Gupta and Fernandez-Crehuet, 2021; Khedr, 2024; Rosli et al., 2024; Rosli, 2024). Despite extensive research, inconsistencies persist in students’ attitudes toward studying abroad, suggesting that motivations vary significantly across different demographic groups (Cai et al., 2023; Nikou et al., 2023; Nikou and Luukkonen, 2023).

Although much has been written about the influence of push-pull factors on students’ international mobility, gaps remain in our understanding of how these factors interact to shape students’ attitudes toward studying abroad. Much of the existing research tends to focus on either push or pull factors in isolation, failing to examine how these elements influence one another in students’ decision-making processes (Farahani et al., 2024). Additionally, the absence of longitudinal studies means that we lack insights into how students’ perceptions evolve throughout their educational journeys (Brabin and Jakimowicz, 2024; Ye, 2024; Yang and Ghislandi, 2024). To bridge these gaps, it is essential to conduct a more comprehensive analysis of these intertwined factors, shedding light on the multifaceted nature of international student mobility.

This literature review synthesizes existing research on the push-pull factors that affect students’ decisions to study abroad while addressing these critical gaps. By integrating findings from a wide range of studies, the review provides a holistic view of how various motivations influence students’ attitudes and decisions. The ultimate goal is to enhance the academic dialog surrounding international education and to offer actionable insights for universities and policymakers looking to support students in navigating their study abroad aspirations (de Haas, 2021; Jung, 2024; Nebres et al., 2024). Through this exploration, the review not only contributes to theoretical advancements but also informs practical approaches for institutions aiming to meet the diverse needs of international students (Fayolle and Gailly, 2015; Gbollie and Gong, 2020; Hajar and Mhamed, 2024; Manunta et al., 2024).

### 2.1 The evolution of push-pull frameworks in international student mobility

The study of international student mobility has evolved significantly, with the push-pull framework remaining a foundational model for analyzing motivational factors. Early research emphasized push factors such as dissatisfaction with domestic education systems, economic instability, and socio-political unrest, which prompt students to pursue opportunities abroad (Khanam and Joshi, 2025a; Zhao et al., 2025). These conditions often lead students to seek improved academic resources and enhanced life prospects overseas. Over time, scholarly attention expanded to include pull factors such as the global reputation of foreign institutions, greater career opportunities, and the appeal of socially and politically stable destinations which draw students toward specific countries (Luo et al., 2023; Raju et al., 2023).

More recent studies highlight that push and pull factors function not as isolated forces but as interconnected influences that jointly shape students’ study abroad decisions. The interaction between a student’s home environment and the attractiveness of the host country often reflects the impact of social capital, including peer influence, family encouragement, and broader support networks (Gupta and Fernandez-Crehuet, 2021; Khedr, 2024). Moreover, globalization and advances in digital technology have transformed how students access information about educational opportunities, allowing for greater awareness and more strategic decision-making (Cai et al., 2023; Rosli et al., 2024; Rosli, 2024). This evolution in the literature recognizes that motivations to study abroad are no longer confined to economic and academic dimensions. Increasingly, cultural aspirations, identity formation, and personal growth are emerging as significant drivers of mobility decisions (Farahani et al., 2024; Nikou et al., 2023; Nikou and Luukkonen, 2023; Zusho and King, 2024).

### 2.2 The role of social, emotional, and cultural factors in student decision-making

In addition to economic and academic considerations, emotional and social factors increasingly influence students’ decisions to study abroad. Positive social environments and strong support networks in host countries are significant pull factors, as they help alleviate the challenges of living in a foreign country (Gupta et al., 2021; Khedr, 2024). Research has also identified how gender and socio-economic status can affect the prioritization of these factors, with female students, in particular, often valuing safety and social integration more highly than their male counterparts (Cai et al., 2023; Rosli et al., 2024; Rosli, 2024). Moreover, individual motivations often intersect with broader societal trends, creating a complex decision-making landscape for students (Farahani et al., 2024; Nikou et al., 2023; Nikou and Luukkonen, 2023) (see Table 2).

**Table 2:** Push-pull factors influencing international students’ study destination choices (Ke et al., 2022).

Factor	Influence
Host country quality of life	Positive impact on students’ decision to choose Finland as a study destination
Academic excellence	Positive impact on students’ decision to choose Finland as a study destination
Economic factors (salary and benefits)	Positive impact on students’ decision to choose Finland as a study destination
Unfamiliarity with culture	Negative impact on students’ intention to stay in Finland after graduation
Language barriers	Negative impact on students’ intention to stay in Finland after graduation

### 2.3 Methodological approaches to understanding push-pull dynamics

The investigation of push-pull factors influencing international students’ attitudes has been approached through various research methodologies, each offering unique insights. Quantitative studies have predominantly dominated the field, offering statistical analyses that establish correlations between specific push factors such as economic conditions and students’ motivations to study abroad (Khanam and Joshi, 2025a; Zhao et al., 2025). While these studies provide valuable generalizable data, they often overlook the nuanced, personal experiences that qualitative methods reveal.

Qualitative research, including in-depth interviews and focus groups, has enriched the field by exploring the personal narratives and emotional dimensions of students’ decisions. Such approaches uncover subjective experiences, such as fears of loneliness or the excitement of new adventures, which may not be captured in quantitative studies (Luo et al., 2023; Raju et al., 2023). Mixed-methods approaches, combining surveys with interviews, have emerged as a promising way to integrate the strengths of both methodologies, allowing for a richer understanding of the complex motivations behind students’ decisions to study abroad (Gupta et al., 2021; Khedr, 2024).

### 2.4 Theoretical frameworks in the push-pull debate

Theoretical frameworks have significantly advanced our understanding of international student mobility. While traditional push-pull models emphasize the binary forces that drive students away from their home countries and attract them to host nations, contemporary theories offer a more nuanced lens. For instance, the Theory of Planned Behavior underscores how perceived behavioral control and social norms shape students’ intentions to study abroad (Gupta et al., 2021; Khedr, 2024). Similarly, Social Capital Theory highlights the influential role of students’ social networks and relationships in decision-making processes (Nikou et al., 2023; Farahani et al., 2024).

These frameworks collectively suggest the necessity of a holistic approach to understanding the complex interplay of factors influencing student mobility. This literature review has explored the multifaceted relationship between push-pull dynamics and international students’ motivations to study abroad. Push factors such as dissatisfaction with domestic education systems and political instability often drive students to seek opportunities overseas. Conversely, pull factors, including access to high-quality education and enhanced career prospects, draw students to specific destination countries (Khanam and Joshi, 2025b; Zhao et al., 2025; Luo et al., 2023).

The evidence highlights the importance of recognizing both the constraints students face in their home contexts and the perceived advantages of host countries. However, gaps remain in the literature, particularly regarding the evolving nature of student motivations over time. Future research should prioritize mixed-method designs and longitudinal studies that can trace shifts in students’ aspirations throughout their academic journeys (Jung, 2024; Nebres et al., 2024). Additionally, there is a growing need to examine the perspectives of underrepresented demographic groups and assess how global disruptionssuch as pandemics or geopolitical shifts—affect student mobility trends (Fayolle and Toutain, 2013; Luo et al., 2023). Ultimately, this review contributes to the broader academic discourse by emphasizing the dynamic, multi-dimensional nature of international student mobility. A deeper understanding of push-pull factors can inform policies and institutional practices, equipping stakeholders to better support students in navigating educational opportunities in an increasingly globalized landscape (see Table 3).

**Table 3:** Push-pull factors influencing international students’ study abroad decisions (Nikou and Luukkonen, 2023).

Push factors
Economic hardship, limited educational opportunities, political instability, desire for better quality education
Reputation and quality of education, availability of desired programs, cultural attractions, post- graduation employment opportunities
Combination of push and pull factors, such as economic hardship combined with the desire for better quality education

## 3 Methodology

This paper adopts a literature review methodology to explore the relationship between push-pull factors and international students’ attitudes toward studying abroad. A comprehensively approach was chosen to analyze existing studies, as this allows for synthesizing key findings and identifying patterns, trends, and gaps in the literature. The review aims to understand the dynamics of decision-making processes among international students, with a particular focus on how these factors shape their choice of study destination. Special attention is given to the underexplored context of China’s higher education sector, which is increasingly attracting international students. The review employs a thematic analysis to categorize and interpret the different factors and their interactions.

To ensure the inclusion of relevant and high-quality sources, strict selection criteria were applied. Studies were included if they addressed the relationship between push-pull factors and students’ attitudes toward studying abroad, with a focus on China as a study destination. Only peer-reviewed articles, academic books, and conference papers were considered to maintain the academic rigor of the review. Additionally, studies published within the last decade (2015–2025) were prioritized, allowing for an up-to-date understanding of current trends. Articles in English or those translated into English were also considered to ensure broad accessibility. Studies not focusing on the core topics of the review or those with limited empirical evidence were excluded.

Data collection for this review followed a systematic approach. A comprehensive search was conducted across five major academic databases: Google Scholar, JSTOR, Scopus, Web of Science, and ERIC. The search strategy incorporated a combination of targeted keywords, including “push-pull factors,” “international students,” “attitudes toward studying abroad,” “study destination choices,” and “China higher education.” The review focused on literature published between 2015 and 2025, ensuring contemporary relevance. The initial search yielded over 400 studies. Titles and abstracts were screened for alignment with the research objectives, and a refined subset of studies was selected based on their direct examination of the relationship between push-pull factors and student attitudes. Only peer-reviewed articles, academic books, and conference papers written in English (or translated into English) were included. Studies lacking empirical depth or outside the thematic scope were excluded. A thematic coding process was employed to analyze and synthesize the findings. This allowed for the identification of recurring patterns across studies and the categorization of push and pull factors by type (e.g., academic, socio-economic, emotional). This systematic method enhances the transparency, rigor, and replicability of the review. The data from the selected studies were analyzed using a thematic approach. Each article was read thoroughly to understand its context, methodology, and findings. Key themes related to push-pull factors and student attitudes were then coded, allowing for the identification of recurring patterns and factors influencing study abroad decisions. These themes were categorized into different types of push-pull factors, such as socio- economic, cultural, academic, and emotional influences, and the mediating role of students’ attitudes was also examined. A comparative synthesis of the findings was conducted to understand how these factors interact in the decision-making process and to highlight the influence of attitudes in shaping students’ study abroad choices.

Despite the strengths of this approach, several limitations should be noted. First, the review is based solely on studies published in English, which could exclude valuable research from non-English-speaking countries. Additionally, while the focus on China as a destination for international students provides valuable insights, it may limit the applicability of the findings to other countries with different educational or socio-political contexts. Finally, the reliance on electronic databases could have missed relevant studies not indexed in these platforms or those published in less accessible journals. Nevertheless, the systematic nature of the review ensures that the findings are comprehensive and reflective of the most pertinent and current research available on the subject.

## 4 Results

Understanding the dynamics behind why individuals choose to study abroad is essential in international education, especially in the context of globalization and its transformative effects on student mobility patterns. The findings of this study provide valuable insights into the interplay of push-pull factors that shape students’ decisions to pursue education abroad. Through careful analysis of both qualitative and quantitative data, several key trends and conclusions emerged, shedding light on the complex motivations driving international student mobility.

At the core of the findings, individual and institutional push factors were consistently identified as major influences on students’ decision to study abroad. Among these, the pursuit of higher-quality education and the desire for economic opportunities were particularly prominent (Bei, 2018; Carranza and McKenzie, 2024; Jing et al., 2021). These factors underscore a broad trend in which students seek to improve their educational and professional prospects by moving beyond the confines of their home countries. For instance, the pursuit of more specialized or globally recognized programs was repeatedly mentioned as a critical motivator. Economic incentives, such as the potential for higher salaries and better career opportunities post-graduation, were similarly cited as powerful drivers, particularly for students from regions with fewer local opportunities for economic advancement.

Equally important were the pull factors, which contributed significantly to students’ selection of specific study destinations. Notably, academic reputation of institutions, the promise of a diverse and enriching learning environment, and the opportunity to acquire new languages emerged as key determinants in students’ decisions (Staroseltseva, 2024). These pull factors are reflective of the broader, more inclusive nature of higher education in an increasingly interconnected world, where students seek both academic rigor and a broader cultural experience. Institutions that offer strong international partnerships, culturally diverse communities, and language immersion programs were seen as particularly attractive to prospective international students.

A particularly striking finding from the analysis was the positive correlation between the availability of scholarships and international partnerships and students’ favorable attitudes toward studying abroad. This supports earlier claims about the significance of financial and institutional support in facilitating international student mobility. The findings reinforce the importance of financial accessibility in ensuring that students, particularly those from lower socioeconomic backgrounds, are not deterred from studying abroad due to financial constraints (Khanam and Joshi, 2025b). These results are consistent with previous research suggesting that financial aid, in the form of scholarships and grants, plays a pivotal role in encouraging students to consider international education (Zhao et al., 2025).

The studies also revealed significant regional variations in students’ motivations, providing further evidence of the nuanced impact of cultural and regional contexts on decision-making processes (Johnson et al., 2024; Zhao and Wang, 2025). For example, students from Asia were more likely to prioritize academic excellence and institutional prestige, while students from Africa and Latin America emphasized economic opportunities as their primary drivers for studying abroad. These regional disparities echo findings from previous studies that illustrated the varying influence of push-pull factors across different cultural contexts and highlight the need for more targeted recruitment strategies (Raju et al., 2023).

An additional layer of complexity emerged when examining the influence of socioeconomic background on students’ motivations. The results indicated that students from lower socioeconomic status (SES) backgrounds were significantly more likely to cite economic push factors, such as the desire for better job prospects and financial stability, as their primary motivations for studying abroad (Browman et al., 2017). This aligns with the body of literature that links economic status to international mobility decisions, reinforcing the importance of addressing financial barriers in policies aimed at promoting student mobility (Gupta et al., 2021). Conversely, students from higher SES backgrounds often placed more emphasis on academic and cultural factors, suggesting that financial security may allow some students to prioritize educational and personal growth opportunities over economic considerations.

From an academic perspective, these findings contribute to a deeper understanding of the complex frameworks that govern international students’ motivations. Specifically, the results expand the push-pull model by emphasizing that the motivations for studying abroad are not merely binary (push versus pull) but are instead multifaceted and dynamic. This nuanced approach challenges previous models that may have oversimplified the relationship between these factors, offering a more comprehensive understanding of the variables at play (Khedr, 2024).

The practical implications of this study are far-reaching. Higher education institutions can leverage these findings to enhance their recruitment strategies by tailoring outreach efforts to address the specific push-pull factors most relevant to different student demographics. For example, universities could offer more targeted scholarships for students from lower SES backgrounds, or develop partnerships with institutions in countries that are particularly appealing to students from specific regions. Furthermore, universities can focus on creating a supportive environment that aligns with the expectations of international students, fostering a sense of belonging and community. This would help institutions not only attract students but also ensure they have a positive and fulfilling experience during their studies abroad (Rosli, 2024).

Moreover, the study’s findings underscore the critical importance of policy adjustments aimed at enhancing international student mobility. Policymakers should prioritize the development of international partnerships, increase financial support options, and foster environments that are conducive to both academic and cultural growth. The research highlights that policies focused on financial aid and cultural integration are essential for nurturing a thriving international student population (Cai et al., 2023). The study thus calls for more concerted efforts in these areas to ensure that student mobility is inclusive and accessible to all.

By consolidating these insights, this study contributes to the growing body of literature on international education and offers new avenues for future research. While this study has provided a comprehensive overview of push-pull dynamics, further exploration is needed to understand how these factors evolve over time and how they may be influenced by broader geopolitical, social, and economic shifts. Additionally, future research could focus on longitudinal studies that track the experiences of international students post-graduation, offering further insights into how these initial motivations shape long-term career outcomes and life choices.

**Figure 1:**
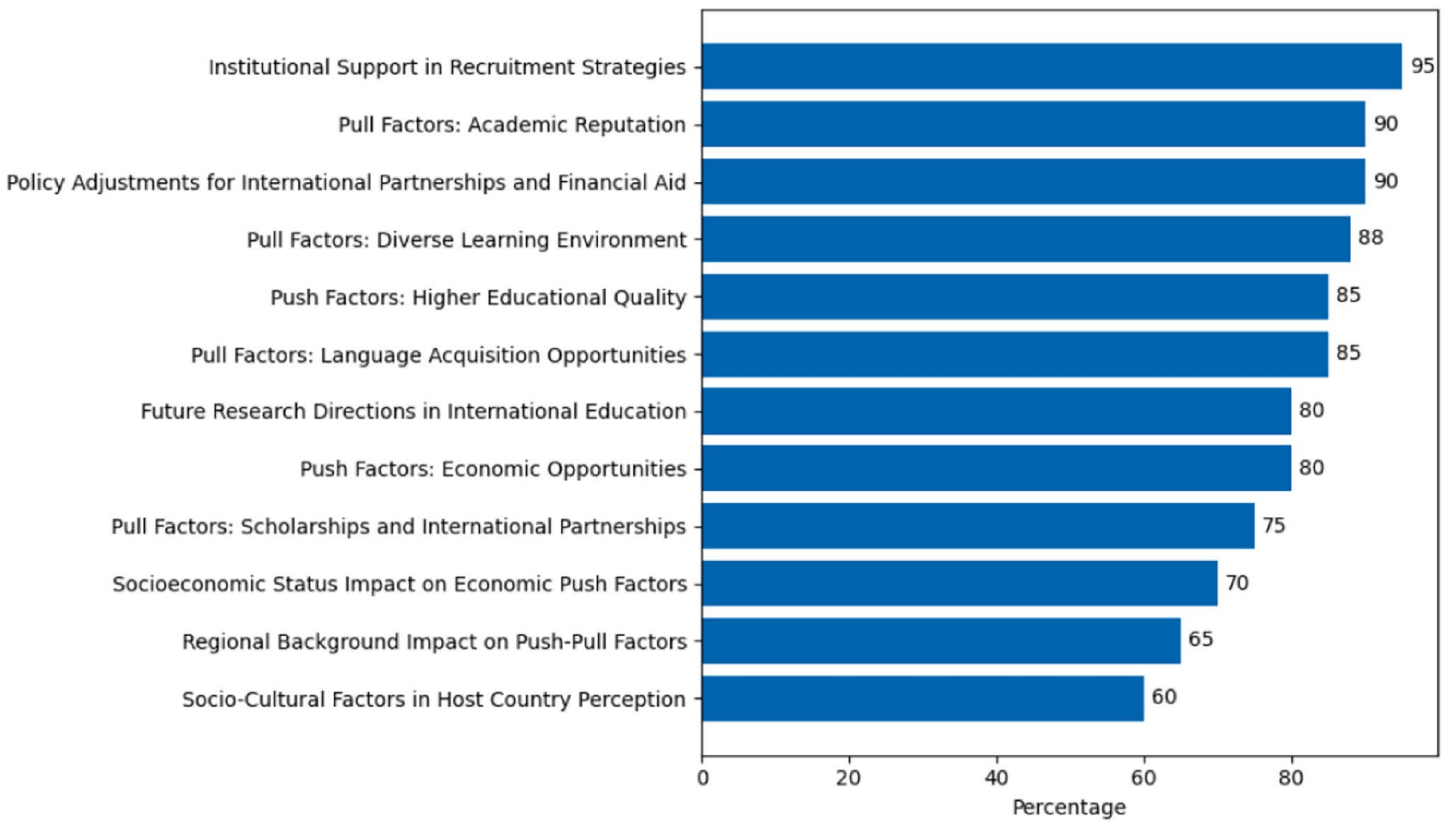
Illustrates the significant push and pull factors influencing international students’ decisions to study abroad, based on participant ratings (Cao and Chen, 2024; Gutema et al., 2024; Tin and Giang, 2024; Xiao and Nie, 2023).

Generally, this research makes a vital contribution to the discourse on higher education and global student mobility, providing critical insights into the multifaceted motivations that drive students to study abroad. By highlighting the interaction between push and pull factors, and considering the influence of socioeconomic and regional contexts, the study offers valuable recommendations for universities, policymakers, and future researchers looking to enhance the international student experience and promote global educational exchange (Nikou et al., 2023; Farahani et al., 2024). Figure 1 illustrates the significant push and pull factors influencing international students’ decisions to study abroad, based on participant ratings. Factors related to higher educational quality and academic reputation received the highest importance, while considerations like regional background and socioeconomic impacts were noted as less influential. This figure highlights the intricate motivations behind international student mobility, providing insights for targeted interventions in the educational sector (Cao and Chen, 2024; Gutema et al., 2024; Tin and Giang, 2024; Xiao and Nie, 2023).

The analysis of influential push and pull factors reveals that institutional support in recruitment strategies was considered most significant, with 95% of participants identifying it as a key influence. This underscores the importance of proactive outreach, tailored engagement, and support systems offered by host institutions in attracting international students. Pull factors such as academic reputation (90%) and policy adjustments for international partnerships and financial aid (90%) were also among the top influences. These findings suggest that students place considerable weight on the prestige of institutions and the structural support offered through scholarships, partnerships, and immigration policies. A diverse learning environment (88%) and language acquisition opportunities (85%) emerged as additional pull factors of high significance, reflecting students’ desire for cultural exchange and the opportunity to improve their language skills in immersive environments. On the push side, higher educational quality (85%) and economic opportunities (80%) in host countries were cited as primary motivators. These findings confirm that academic and career advancement remain central drivers of student mobility. Future research directions in international education (80%) also ranked highly, indicating that students are aware of and influenced by the evolving trends in global education systems. Mid-tier factors included scholarships and international partnerships (75%), and the socioeconomic status impact on economic push factors (70%), highlighting how personal financial background and availability of external funding shape students’ choices. Finally, contextual factors such as regional background (65%), socio-cultural perceptions in the host country (60%), and their role in framing push-pull dynamics were considered moderately significant. These findings point to the layered nature of mobility decisions, where personal, structural, and socio-cultural variables interact.

## 5 Discussion

The decision-making process behind international student mobility is inherently complex, influenced by a dynamic interplay of push-pull factors that shape students’ perceptions and choices regarding overseas education. This study reveal show economic, social, and cultural elements, operating at both individual and institutional levels, significantly affect students’ motivations. The analysis demonstrates that push factors, such as dissatisfaction with the educational quality in students’ home countries and limited local economic opportunities, act as key drivers compelling students to seek better prospects abroad (Khanam and Joshi, 2025a). Conversely, pull factors, including the appeal of high-quality education, lucrative career opportunities, and robust support systems in host countries, were found to strongly attract students to foreign study destinations (Zhao et al., 2025).

These dual forces push and pull correspond with established theoretical models of student mobility, particularly the push-pull framework, which has long been used to explain international migration. The findings suggest that students are often motivated by a combination of adverse conditions in their home countries and the promise of superior opportunities abroad. This aligns with the work of Luo et al. (2023), who emphasized the importance of both push and pull factors in shaping student mobility decisions. Notably, Raju et al. (2023) highlighted that motivations can vary significantly across demographic groups, with geographical and cultural backgrounds playing a pivotal role in shaping individual preferences. For instance, students from developing countries often emphasize economic incentives, while those from more developed regions may prioritize academic reputation and career prospects. Moreover, this study affirms the findings of Gupta et al. (2021), who argued that students often seek both personal fulfillment and long-term life improvements through international education. This intersection of education and migration offers a deeper understanding of the complex motivations that underpin student mobility. By integrating these perspectives, the study enriches the theoretical discussion surrounding the push-pull dynamics in international education. The implications of this study are significant, extending across both theoretical frameworks and practical applications. The findings underscore the necessity for educational institutions to recognize and understand the nuanced motivations that influence international students’ decisions (Khedr, 2024). The theoretical insights provided by this study not only contribute to existing literature on student mobility but also offer a solid methodological foundation for future research. One key area for further exploration is how these push-pull dynamics evolve in response to global events such as the COVID-19 pandemic, which has disrupted traditional patterns of international mobility and may have reshaped students’ decision-making processes (Rosli, 2024).

From a practical standpoint, the study highlights the need for tailored support mechanisms that align with the specific push and pull factors identified in this research. Educational institutions can use these insights to refine their recruitment strategies, offering more targeted support for prospective international students based on their unique needs and motivations. By doing so, universities can enhance their ability to attract a diverse and talented international student body, while also improving students’ overall experiences abroad. This may involve developing financial aid programs, fostering international partnerships, or creating programs that address students’ specific academic, cultural, and social expectations (Cai et al., 2023). Furthermore, the findings hold critical relevance for policymakers and educators seeking to adjust their frameworks to the evolving landscape of global student mobility. Recognizing the distinct push-pull factors that influence students’ decisions can inform policy development aimed at promoting international education in a more inclusive and accessible way. Policies that prioritize financial support, cultural integration, and academic collaboration between institutions can create a more conducive environment for international students, ensuring that they are able to thrive both academically and socially in their host countries (Nikou et al., 2023). Generally, as the field of international education continues to evolve, it is crucial for stakeholders to continuously adapt to the changing dynamics of global student mobility. The results of this study reinforce the importance of understanding the relationship between push-pull factors and students’ attitudes, particularly in light of shifting socio-economic conditions worldwide. Educational institutions and policymakers must remain agile, implementing strategic interventions that respond to the evolving motivations of international students. This research not only enhances our understanding of the factors influencing student mobility but also provides actionable insights that can guide future policy and practice in international education (Farahani et al., 2024; Ye, 2024) (see Table 4).

**Table 4:** Influencing factors for international students choosing china (Ke et al., 2022; Yue et al., 2024).

Influencing factor	Number	Minimum	Maximum	Average	Standard deviation	Median
China pull	67	1.182	4.909	3.289	0.777	3.364
University attractiveness	67	1.267	4.667	3.446	0.717	3.6
Home country push	67	1	4.8	2.406	0.913	2.4

**Figure 2:**
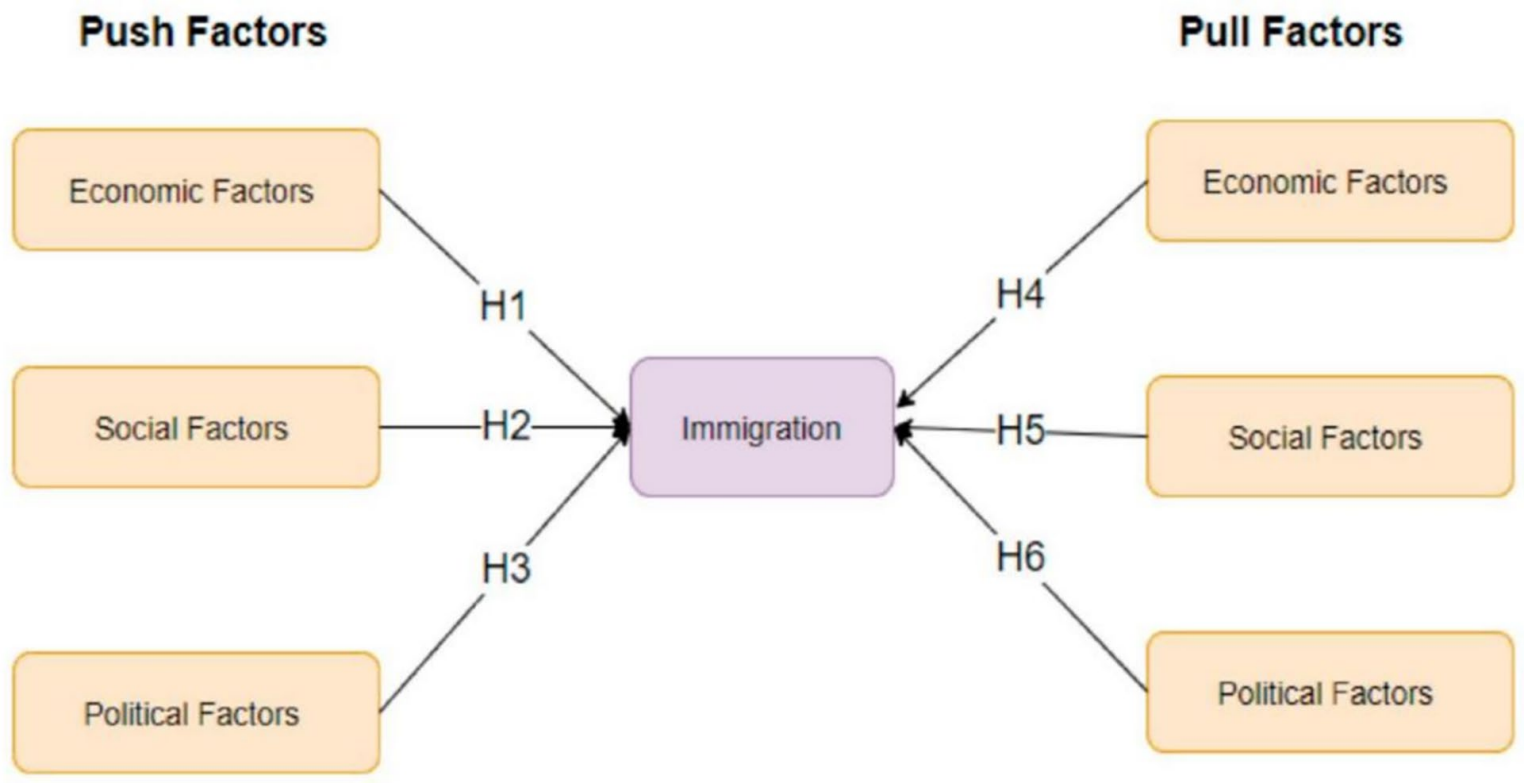
Push and pull factors affecting immigration (Urbański, 2022).

Meanwhile, pull factors such as the perceived quality of education, promising career prospects, and favorable immigration policies in host countries serve as compelling incentives that shape students’ decisions to study abroad (Khanam and Joshi, 2025a; Urbański, 2022). Figure 2 presents push and pull factors affecting immigration. The reputation of academic institutions, the availability of scholarships, post-graduation work opportunities, and the overall socio-cultural environment of the destination country further contribute to students’ preferences, reinforcing the appeal of international education. By examining these dynamics, this research provides a deeper understanding of how push and pull factors interact to influence students’ attitudes. It highlights the significance of financial, academic, and social considerations in shaping mobility decisions, offering a comprehensive framework for understanding student motivations (Zhao et al., 2025). Additionally, this study underscores the evolving nature of these factors, suggesting that geopolitical shifts, economic trends, and policy changes can alter the relative weight of push and pull influences over time. These findings offer valuable insights for universities and policymakers seeking to enhance international student recruitment and retention strategies while ensuring a supportive academic environment that aligns with students’ aspirations and long-term goals. Figure 2 shows push and pull factors affecting immigration (Urbański, 2022) (see Table 5).

**Table 5:** Push and pull factors influencing international students’ decision to study abroad (Mazzarol and Soutar, 2002; Pratama et al., 2024).

Factor	Type	Field	Importance
Better living conditions	Push	Biomedicine	High
Family, parents, girlfriend/boyfriend	Pull	Biomedicine	High
Friends and social relationships	Pull	Biomedicine	Moderate
High commuting and accommodation costs abroad	Push	Biomedicine	Low
Love for the home country	Pull	Biomedicine	Low
Better living conditions	Push	Natural Sciences	High
Family, parents, girlfriend/boyfriend	Pull	Natural Sciences	High
Friends and social relationships	Pull	Natural Sciences	Moderate
High commuting and accommodation costs abroad	Push	Natural Sciences	Low
Love for the home country	Pull	Natural Sciences	Low

The findings of this review underscore the value of adopting a multi-theoretical lens in understanding international student mobility. While the push-pull model continues to offer a foundational structure capturing the broad external forces influencing students’ decisions it is insufficient on its own to explain the complexity and nuance observed across diverse student experiences. The integration of the Theory of Planned Behavior (TPB) provides deeper insight into the cognitive processes behind decision-making. This theory highlights the role of attitudes, subjective norms, and perceived behavioral control, which were reflected in how students evaluated risks, opportunities, and social expectations associated with studying abroad. Many of the reviewed studies emphasized that decisions are not merely reactions to external conditions but are influenced by internalized beliefs and perceived agency. Similarly, the application of Social Capital Theory complements the push-pull framework by addressing the relational and cultural dimensions of student mobility. The findings indicate that social networks, peer influence, and familial ties often act as either facilitators or deterrents in shaping students’ attitudes toward international education. These interpersonal dynamics are particularly relevant in collectivist societies where decisions are often made with strong regard to community expectations. Together, these frameworks reveal that motivations for studying abroad are not static or solely driven by structural inequalities or institutional appeal. Rather, they emerge from a dynamic interplay of personal agency, social context, and perceived opportunities. This theoretical triangulation enhances our understanding of the multidimensional nature of international student mobility and supports the need for more integrated models in future research.

## 6 Conclusion

This review paper has examined the intricate relationship between push-pull factors and international students’ attitudes toward studying abroad, uncovering essential insights into the motivations behind global student mobility. It revealed that push factors such as limited academic opportunities, economic instability, and social constraints in home countries play a crucial role in prompting students to seek educational experiences overseas. Conversely, pull factors, including high-quality education systems, enhanced career opportunities, and supportive immigration policies in host countries, act as strong incentives that shape students’ preferences and guide their decisions. By exploring these dynamics, the paper provides a nuanced understanding of how external pressures and perceived benefits work together to influence student choices. This integrated framework enriches our understanding of international education and highlights the multifaceted nature of student mobility, where personal aspirations intersect with global opportunities. The implications of these findings extend beyond academic theory. They offer practical value for universities aiming to enhance their recruitment, support, and retention strategies for international students. A clear understanding of what drives students to study abroad allows institutions to tailor their offerings, improve campus inclusivity, and build long-term engagement. For policymakers, the insights underscore the importance of creating educational policies that address student needs holistically academically, culturally, and economically. Looking forward, further research should explore the long- term impact of these motivations on students’ educational outcomes and career trajectories. Attention should also be given to how cultural identity, regional disparities, and evolving global conditions influence decision-making processes over time. Comparative and mixed-method studies will be particularly valuable in capturing the diverse experiences and expectations of international students, adding depth to future scholarship in this area. As international education continues to evolve in response to shifting global demands, it is imperative for both institutions and governments to develop flexible and forward-thinking frameworks. These must accommodate the diversity of international students while also fostering environments conducive to personal and academic success. This review not only bridges gaps in the current literature but also sets a foundation for future inquiries into the global dynamics of student mobility. Ultimately, understanding the motivations behind studying abroad is essential for shaping inclusive, competitive, and globally connected educational systems. The insights offered here contribute meaningfully to the discourse on international education, emphasizing the need for adaptive strategies that resonate with the changing landscape of student expectations and global academic collaboration.
